# Super-Resolution Imaging at Mid-Infrared Waveband in Graphene-nanocavity formed on meta-surface

**DOI:** 10.1038/srep37898

**Published:** 2016-11-29

**Authors:** Jingzhong Yang, Taisheng Wang, Zuolong Chen, Bingliang Hu, Weixing Yu

**Affiliations:** 1 State Key Laboratory of Applied Optics, Changchun Institute of Optics, Fine Mechanics & Physics, Chinese Academy of Sciences, No.3888, Dongnanhu Road, Changchun, Jilin, P. R. China; 2 University of the Chinese Academy of Sciences, Beijing, 10039, P. R. China; 3 Troop 63861, People’s Liberation Army of China, Baicheng 137001, P. R. China; 4 Key Laboratory of Spectral Imaging Technology, Xi’an Institute of Optics and Precision Mechanics, Chinese Academy of Sciences, No.17, Xinxi Road, Xian 710119, P. R. China

## Abstract

Plasmonic structured illumination microscopy (PSIM) is one of the promising wide filed optical imaging methods, which takes advantage of the surface plasmons to break the optical diffraction limit and thus to achieve a super-resolution optical image. To further improve the imaging resolution of PSIM, we propose in this work a so called graphene nanocavity on meta-surface structure (GNMS) to excite graphene surface plasmons with a deep sub-wavelength at mid-infrared waveband. It is found that surface plasmonic interference pattern with a period of around 52 nm can be achieved in graphene nanocavity formed on structured meta-surface for a 7 μm wavelength incident light. Moreover, the periodic plasmonic interference pattern can be tuned by simply changing the nanostructures fabricated on meta-surface for different application purposes. At last, the proposed GNMS structure is applied for super-resolution imaging in PSIM and it is found that an imaging resolution of 26 nm can be achieved, which is nearly 100 folds higher than that can be achieved by conventional epi-fluorescence microscopy. In comparison with visible waveband, mid-infrared is more gently and safe to biological cells and thus this work opens the new possibility for optical super-resolution imaging at mid-infrared waveband for biological research field.

In last few decades, the great progress in optical imaging has led to a profound impact on biological living cells research. Various methods were developed to improve the optical imaging resolutions^1^,^2^ including stimulated emission depletion microscopy (STED)^3^,^4^, near-field scanning optical microscopy (NSOM)^5^,^6^, and structured illumination microscopy (SIM)^7^,^8^, etc. Among these methods, more and more attentions are being paid to SIM for it can achieve high resolution optical imaging at wide-field. However, the spatial resolution of SIM is theoretically limited to ∼*λ*/4*NA*, where *λ* is the wavelength of incident light and *NA* is numerical aperture of the objective lens^9^,^10^. More recently, plasmonic structured illumination microscopy (PSIM) was proposed to further improve the resolution by taking advantage the higher wave vector of surface plasmons^11^,^12^. Normally, noble metal is used for generating surface plasmons. However, the main problem of noble metals is that the loss is rather high so that the coupling efficiency as well as the propagation length of surface plasmons is limited.

Graphene, as a two-dimensional (2D) material formed by carbon atoms in honeycomb arrangement^13^, is only about 0.34 nm thick and has drawn extensive attentions in recent years. More recently, it was reported that strong coupling of light with electrons in graphene, i.e. graphene plasmons^14^,^15^,^16^, at middle-infrared and THz wavebands can be realized, and thus magnetic (TM) polarized surface plasmons can be stimulated and excited^17^,^18^. In comparison with conventional plasmons excited in noble metals, graphene plasmons have stronger ability to confine optical field with lower loss. Moreover, it can be tuned by gating or doping to make active devices in a broadband from middle-infrared to THz due to its extreme high conductivity^19^,^20^. Therefore, there is the possibility to incorporate graphene plasmons in PSIM method to further improve the optical imaging resolution.

In this work, we propose a so called graphene nanocavity on meta-surface (GNMS) structure model by intelligently integrating graphene nanocavity with meta-surface for super-resolution imaging in PSIM method. The dispersion relationship of the model is firstly discussed and then followed by simulating the structure by employing FDTD method. It is found that the plasmonic interference pattern generated on graphene has a period of 52 nm for a 7 μm incident light wavelength. There have been PSIM techniques based on surface plasmons excited on the surface of thin silver, and the imaging resolution improvement with 3 to 4 folds in comparison with conventional epi-fluorescence at visible waveband have been reported^11^,^21^,^22^. Nevertheless, once the model is applied for PSIM for optical imaging and it is found a resolution of 25 nm can be achieved, which is about 100 folds higher than that of traditional optical imaging and thus has the great potential to be applied for optical super-resolution imaging for biological research.

## Results

### Structure description and analytical theory

**Figure 1:**
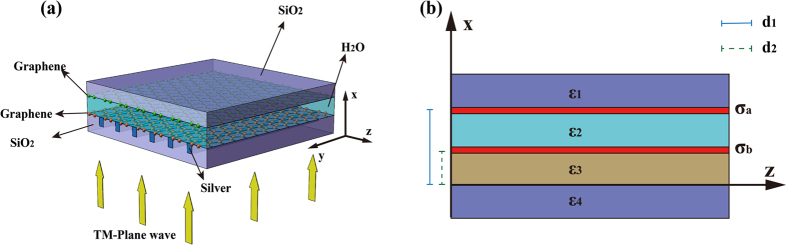
Schematic of GNMS. (**a**) Schematic of the GNMS model which consists of the nanocavity formed by two layers of graphene located on the top of a meta-surface, and the meta-surface is formed in a thin silver layer. (**b**) The cross-sectional view of the GNMS model.

Figure 1 shows the proposed GNMS model that consists of a nanocavity formed by two layers of graphene^23^ located on the top of a meta-surface structure, in which the wave vector of certain scattered or diffracted light by the silver grating matches with the graphene plasmonic wave vector under the phase-matching condition^24^,^25^. Additionally, TM polarized light incidents normally onto the silver grating to illuminate the structure from the bottom. Figure 1(b) shows the simplified cross-sectional view of the model to assist analyzing its electromagnetic property.

According to Maxwell’s electromagnetic theory, the H and E field in each layer of GNMS can be expressed.

In Layer 1,


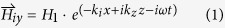



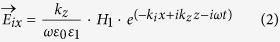



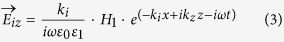


In Layer 2 and 3,


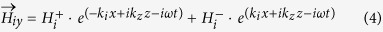



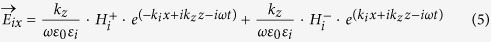



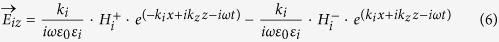


In Layer 4,


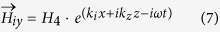



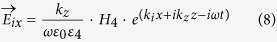



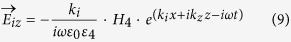


Here, 
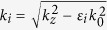
 with *i* = 1–4 stands for the wavenumber along x direction in each layer. *ω* is the angular frequency, *ε*_*i*_ is the relative permittivity of the materials in layer *i*, and *k*_0_ *i*s the free-space wave vector. Under the assumption that surface current exists on graphene, one can obtain a series of relationships by matching the boundary conditions at each interface.

At interface *x* = *d*_1_, since the boundary condition is 
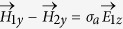
, 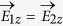, therefore one can get,


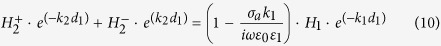



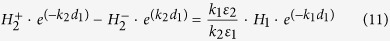


By combining equations (10) and (11), one can get,


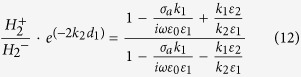


Similarly, the relationships between layer 2 and 3 (*x* = *d*_2_) can be derived by considering boundary conditions of 
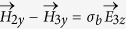
, 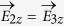,


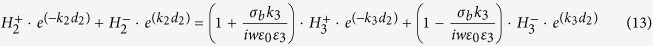



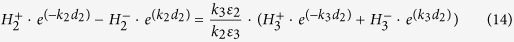



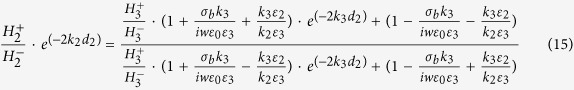


Because there is no surface current exists at the interface between layer 3 and 4 (*x* = 0) and the boundary conditions are 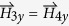, 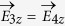, hence one can get,


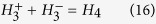



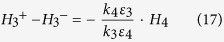



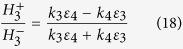


Finally, the following dispersion relationship can be obtained,


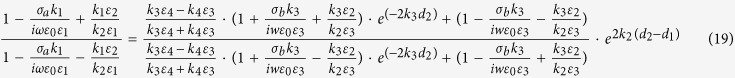


In GNMS model, both *ε*_1_ and *ε*_4_ are the relative permittivity of silica, and *ε*_2_ is the relative permittivity of water. Once the duty cycle of silver gate is set as *γ*, the relative permittivity of dielectric 3 is determined to be 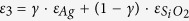 approximately. Besides, the dispersion relationship is also related to the permittivity of each layer determined by the angular frequency of incident light. Therefore, the wave vector (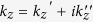) of surface plasmons on graphene can be calculated.

**Figure 2:**
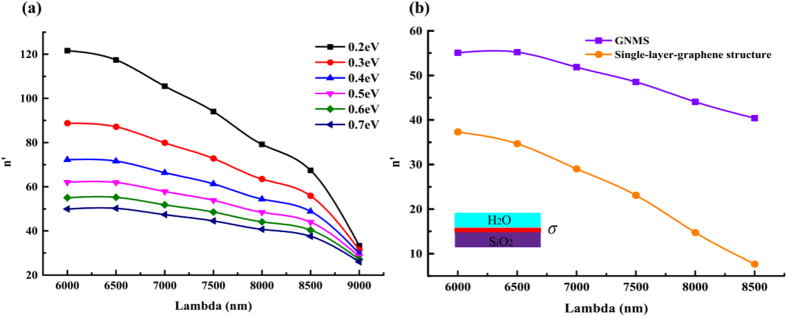
Dispersion curve of GNMS. (**a**) Dispersion relationship of GNMS under different chemical potential of graphene. (**b**) Dispersion curve of GNMS and single-layer-graphene structure (shown in the inset picture) when chemical potential of graphene is 0.6 eV.

Figure 2(a) shows the calculated dispersion relationship of GNMS under different chemical potential of graphene. In the figure, *n*′ is defined by 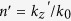, which shows the ratio of wave vector between graphene plasmons and the free space incident light. If *n*′ is much larger than 1, it means that the graphene plasmonic wave with a much higher wave vector has been obtained, which is preferred for PSIM. In the calculation, the thickness of silver grating is *d*_2_ = 50 *nm*, and the thickness of water Δ*d* = *d*_1_−*d*_2_ = 10 nm. The conductivity of graphene σ can be determined by Kubo formula^26^,^27^,^28^,^29^,


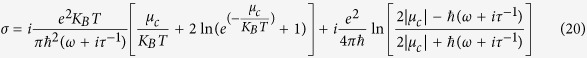


Where *e*, *ω*, *T*, *μ*_*c*_, *τ*, *k*_*B*_ and *ħ* stands for the charge of electron, radian angular frequency, temperature, chemical potential, momentum relaxing time, Boltzmann constant and the reduced Planck constant respectively. The first term of equation (20) represents the intraband conductivity and the second term is the interband conductivity approximately. Furthermore, equation (20) can be simplified to Drude-like form when the intraband term domains on the condition that *ħω* *<* *μ*_*c*_, *K*_*B*_*T* and *μ*_*c*_>>*K*_*B*_*T*^30^,^31^,^32^,


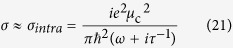


When *ω*<*ω*_*oph*_ (*ω*_*oph*_ is the optical phonon frequency)^33^, the relaxing time *τ* can be calculated by 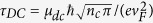
34,35,36, where *n*_*c*_, *μ*_*dc*_ and *v*_*F*_ stands for electron concentration, carrier mobility and the Fermi velocity respectively. If we define 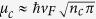, then the conductivity of graphene can be expressed as,


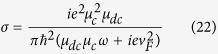


In this paper, the carrier mobility Fermi velocity is set as *v*_*F*_ = 10^6^ *m*/*s* and carrier mobility is *μ*_*dc*_ = 10000 *cm*^2^*V*^−1^*s*^*−1*^.

For comparison, the dispersion curve of single-graphene structure (shown in the inset in Fig. 2(b)) is given as well. The dispersion relationship of single-layer-graphene structure can be expressed as^35^,^37^,^38^.


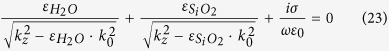


It can be found from Fig. 2(b) that the wave vector of graphene plasmons of GNMS is apparently larger than that in single-layer-graphene structure. Therefore, the GNMS model is preferred for PSIM for optical imaging resolution enhancement purpose.

### Simulation results

**Figure 3:**
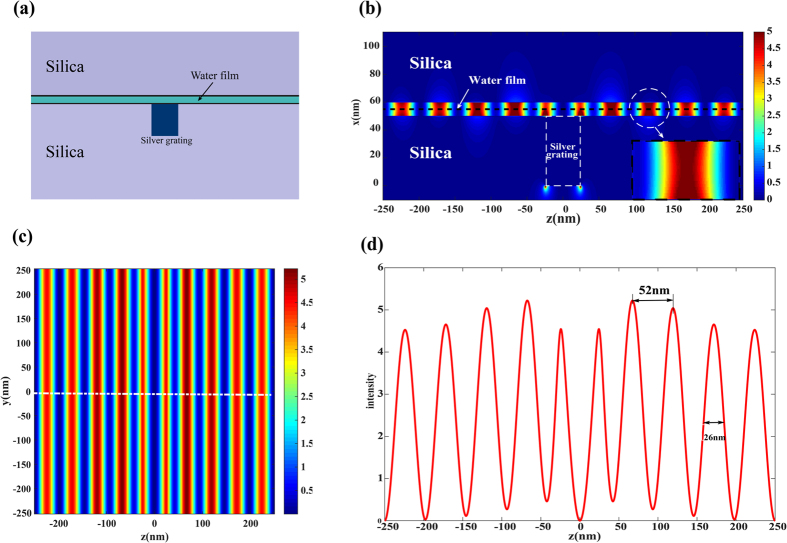
Simulation of GNMS structure and results. (**a**) Cross-sectional view of the GNMS structure. (**b**) Cross-sectional view of electric field distribution of graphene plasmonic interference pattern. (**c**) Top view of electric field distribution of graphene plasmonic interference pattern. (d) Electric field distribution along the white dashed line in Fig. 3(**c**).

To further study the electromagnetic property of GNMS structure, the finite-difference time domain (FDTD) method was employed to simulate the GNMS structure (See Methods Section). Figure 3 shows the simulation results of 20 *um* × 20 *um* GNMS structure. In the simulation, the thickness of the silica substrate is set as *d*_*s*_ = 150 *nm* and two dimensional periodic rectangular slits array with depth of 50 nm and period of 500 nm is fabricated in silica substrate. The slits are then filled with silver to form a two dimensional periodic silver grating with a period of 500 nm and a duty cycle of 0.1. This meta-structure is used to stimulate graphene plasmons. The nanocavity in between two layers of graphene film has a thickness of 10 nm and is filled with water. The top and bottom graphene film is supported by silica substrate and meta-surface respectively. The silica substrate on the top graphene film has a thickness of 100 nm. The wavelength of the incident light is set as 7 μm, corresponding with angular frequency of around 2.69 × 10^14^ *rad*/*s*. Actually, the wavelength of incident light can be tuned in infrared waveband and the proposed structure performs well in waveband ranging from 6 μm~9 μm. The relative permittivity of silica, water and silver is exported from FDTD material explorer and is 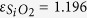, 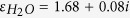, *ε*_*Ag*_ = −1634.61 + 566.379*i*. The chemical potential, carrier mobility and Fermi velocity of graphene is set as: *μ*_*c*_ = 0.64 *eV*, *μ*_*dc*_ = 10000 *cm*^2^V^−1^, *v*_*F*_ = 10^6^ *m*/*s* respectively. Figure 3(a) shows cross sectional view of GNMS structure and Fig. 3(b) shows the cross sectional view of electric field distribution of the formed periodic plasmonic interference pattern in GNMS structure in z-x plane. As can be seen from Fig. 3(b), the electric field distribution is quite uniform inside the graphene nanocavity. This means if the observed object is put into the nanocavity, the object is always within the working depth of PSIM. Figure 3(c) shows the electric field distribution of plasmonic interference pattern at plane *x* = 55 *nm*. Figure 3(d) is the electric field distribution along the dashed line. As can be seen, the intensity of electric field in graphene nanocavity is strongly enhanced which is about 5 times of that of the incident light. The period of the graphene plasmonic interference pattern is 52 nm and the full width at half maximum (FWHM) is around 26 nm. Considering the wavelength of the incident light is 7 μm and the wavelength of graphene plasmonic wave is calculated to be 104 nm, hence the wave vector of graphene is increased for about 67 folds higher in graphene nanocavity.

**Figure 4:**
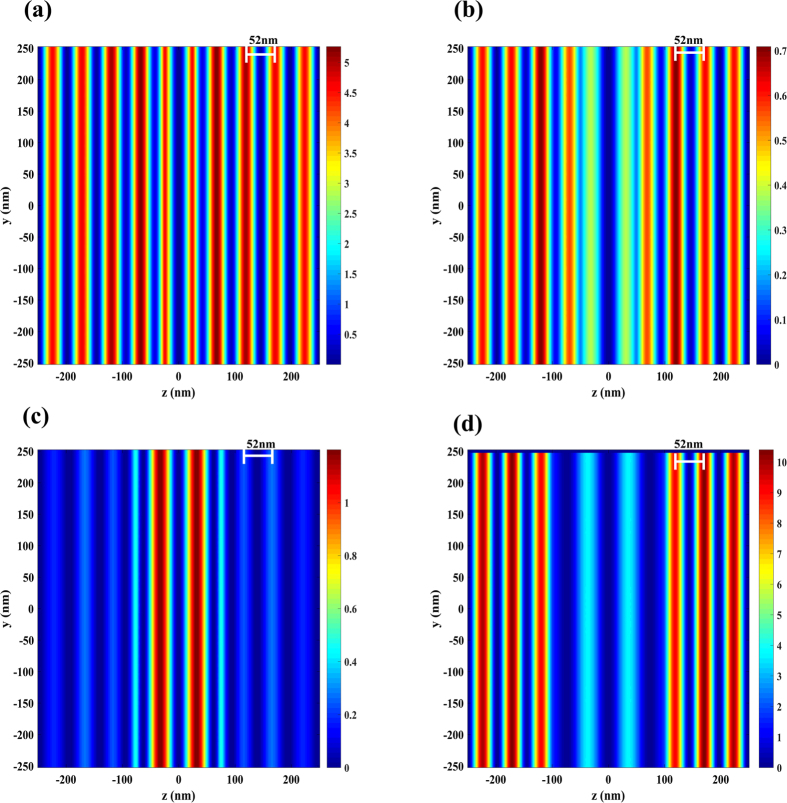
The graphene plasmonic interference pattern with different duty cycle of grating. (**a**) Interference pattern with duty cycle is set as 0.1. (**b**) Interference pattern with duty cycle is set as 0.2. (**a**) Interference pattern with duty cycle is set as 0.3. (**a**) Interference pattern with duty cycle is set as 0.4.

To explain the function of the grating in meta-surface on the graphene plasmonic interference pattern, parameters of the silver grating including duty cycle, material and period are discussed respectively. Figure 4 shows the influence of the grating duty cycle on graphene plasmonic interference pattern. Obviously, the grating duty cycle ranging from 0.1–0.4 has little impact on the field distribution in nanocavity as the period of the plasmonic interference pattern is always 52 nm. However, it is found that the uniform of the field distribution becomes worse when duty cycle larger than 0.1. Furthermore, the structure with duty cycles of 0 and 1 was simulated as well and it is found that in graphene plasmons can not be excited for both cases, which means that the grating structure is necessary to stimulate graphene plasmons.

In order to show the effect of the grating material on graphene plasmons, grating structure with different materials was studied and the results are shown in Table 1(a). As can be seen clearly that although the grating material is changed, but the period of the plasmonic interference pattern keeps constant. This means graphene plasmons can always be excited for different grating materials. However, the peak intensity of graphene plasmons is significantly different for different material. As can be seen, the peak intensity for metal and semiconductor materials is about the same. But for dielectric material, i.e. Al_2_O_3_, the peak intensity is quite weaker. We attribute this reason to the possible contribution localized surface plasmonic enhancement effect of metal and semiconductor materials.

Furthermore, the effect of the period of metal grating on graphene plasmons was also discussed for the same duty cycle of 0.1 and the results are shown in Table 1(b). It is found that the period of the silver grating seems has no effect on the period of the graphene plasmonic interference pattern as it is always 52 nm when grating period changes from 500 nm to 1000 nm. However, the peak intensity of the field is significant different. The maximum intensity can be obtained when grating period is 700 nm. Consequently, in the next analytical calculation, the parameter *γ* will be set as 0 approximately for accuracy when the grating duty cycle is small.

### Tuning of graphene plasmons in nanocavity

To tune the graphene plasmons in nanocavity, the influence of the structural parameters on the graphene plasmonic interference field is systematically studied. The structural parameters that can be changed include the thickness of water film Δ*d* and the height of silver grating *d*_2_. Table 2(a) shows the period of the plasmonic interference pattern against the thickness of water film when silver film thickness is fixed to be 50 nm. The propagation length of the graphene plasmons (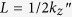) was also calculated and is shown in the same table. In general, it is found that the smaller the water film thickness, the smaller the period of the plasmonic interference pattern. Meanwhile, the propagation length of graphene plasmons becomes smaller when the water film thickness becomes smaller. Table 2(b) shows the influence of silver film thickness on graphene plasmons. As can be seen clearly, despite the plasmonic wavelength have relationship with parameter *d*_2_ through the dispersion equation (19), it seems that the film thickness of the silver grating almost does not influence graphene plasmons when thickness changes from 40 to 80 nm, which well verified the previous assumption.

Moreover, it is found that the period of graphene plasmonic interference pattern can also be tuned by changing the chemical potential of graphene. Table 2(c,d) shows the period of the plasmonic pattern against the chemical potential of the graphene film on top and bottom respectively. In general, the lower the chemical potential, the smaller the period of the plasmonic pattern.

### Application of GNMS structure for super-resolution imaging

**Figure 5:**
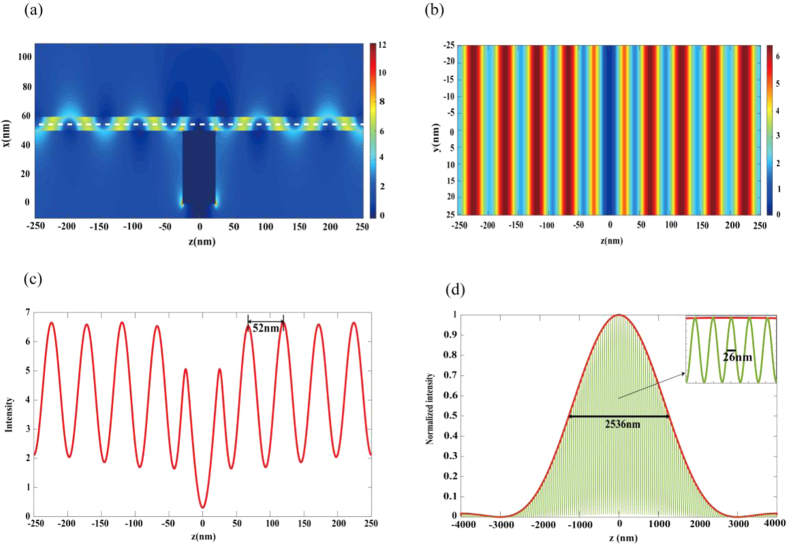
Application of GNMS structure in super-resolution imaging. (**a**) Cross sectional view of electric field distribution of surface plasmonic interference pattern. (**b**) Top view of the graphene plasmonic interference pattern in water in nanocavity. (**c**) Electric field intensity distribution along the dashed line in Fig. 5(b). (**d**) Comparison of the normalized FWHM of a quantum dot obtained by conventional epifluorescence microscopy (red line) and by using GNMS in PSIM (green line). The inset shows the normalized FWHM in Fig. 5(d)

The proposed GNMS structure can be applied for super-resolution imaging at mid-infrared waveband. If DNA or protein is put in the nanocavity, the formed plasmonic pattern can be used to illuminate the biological sample as a structured illumination light for PSIM method. Figure 5(a–c) show the simulation results of the electric field distribution of graphene plasmonic interference pattern. According to the theory of PSIM^11^,^12^, the resolution limit of optical imaging is determined by *λ*_*emission*_/(2*NA*+2*NA*_*effective*_), where *λ*_*emission*_ is the wavelength of incident light and *NA*_*effective*_ represents 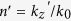. which means the larger the *NA*_*effective*_, the higher resolution limit the PSIM can be achieved. Since the period of the plasmonic pattern is 52 nm, the resolution limit can be achieved is 25 nm for PSIM. Figure 5(d) shows the FWHM of the point spread function (PSF) of a traditional immersion oil objective lens with a NA of 1.42 and the reconstructed PSF of GNMS structure based PSIM for a 10 nm quantum dot (See Methods Section). As can be seen, the FWHM is about 2536 nm for traditional optical lens and therefore the imaging resolution has been enhanced for more than 100 folds. This has significantly improved the optical imaging resolution at mid-infrared waveband.

## Discussion

As a summary, we have proposed a so called GNMS model for PSIM method to improve the optical imaging resolution. The GNMS model takes advantage of merits of both graphene and meta-structure. As proved by both analytical and numerical simulations, deep compression of the period of plasmonic interference pattern can be achieved in graphene nanocavity at mid-infrared waveband. Moreover, the proposed GNMS model can be applied for optical imaging resolution when applied in PSIM method. The rigorous numerical simulation results show that the imaging resolution has been enhanced for more than 100 folds by GNMS in comparison with traditional optical imaging method. More importantly, as the infrared light is safer than visible light for biological samples, the proposed GNMS method should be more preferred for biological research when applied in optical imaging.

## Methods

The proposed GNMS is simulated by using Finite Difference Time Domain (FDTD) software supported by Lumerical Solutions Inc. In the simulation, temperature is set as 300 K, the model of graphene is represented by a 2D-rectangle to reduce the simulation time and ensure the precision of the simulation. The scattering rate and chemical potential of graphene are set according to the design, and the conductivity scaling of graphene keeps 1. Furthermore, in the simulation the 8^th^ mesh accuracy which is the highest is used. The mesh type is set as auto-uniform which means that the minimum grid is the 34^th^ of the minimum wavelength. The periodical boundary condition is set along both y and z axes and PML boundary condition is used in x direction. In addition, in the region of water film and silver grating, a finer grid of 0.5 × 5 × 0.5 *nm*^3^ is used to ensure the sufficient precision near graphene regions.

In the numerical experiment of super-resolution imaging, a 10 nm quantum dot is used as the object. The PSF in far field was calculated by the first order Bessel functions^39^,


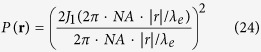



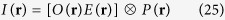


Where *J*_1_ is the first-order Bessel function, NA is the numerical aperture of oil objective, *λ*_e_ represents the reflection wavelength of the quantum dot and is the radius of quantum dot, thus in the far field, the image of the quantum dot can be obtained. For GNMS based PSIM method, *O*(**r**) is set as 1 at where the dot is illuminated. Furthermore, at least three different phase, i.e. 0, 120°, −120°, of the illumination pattern should be recorded to realize the image reconstruction. This can be achieved by changing the angle of the incident light or by employing the vortex beams with different topological charges.

## Additional Information

**How to cite this article**: Yang, J. *et al.* Super-Resolution Imaging at Mid-Infrared Waveband in Graphene-nanocavity formed on meta-surface. *Sci. Rep.* **6**, 37898; doi: 10.1038/srep37898 (2016).

**Publisher's note:** Springer Nature remains neutral with regard to jurisdictional claims in published maps and institutional affiliations.

## Figures and Tables

**Table 1:** Effects of grating parameters on graphene plasmons.

(a)						
Material of grating in meta-surface	Ag	Au	Al	Si	Al_2_O_3_	
Period of surface plasmonic interference pattern (nm)	52.0	52.0	52.0	52.0	52.0	
Peak intensity of surface plasmonic interference pattern	4.56	4.14	4.13	4.55	0.32	
**(b)**						
Period of silver grating (nm)	500	600	700	800	900	1000
Period of surface plasmonic interference pattern (nm)	52.0	52.0	52.0	52.0	52.0	52.0
Intensity of surface plasmonic interference pattern	4.56	5.96	36.04	10.59	4.33	1.24

(a) Material; (b) Period.

**Table 2:** Tuning of the period of graphene plasmonic interference pattern.

**(a)**						
Thickness of water film (nm)	5	8	11	14	17	20
Period of surface plasmon interference pattern by analytical calculation (nm)	38.6	47.8	55.0	61.0	66.2	70.8
Period of surface plasmon interference pattern by numerical simulation (nm)	38.0	48.0	54.0	60.0	65.5	71.0
Propagation length of the graphene plasmons by analytical calculation (nm)	231.2	287.9	332.6	369.6	401.3	428.7
**(b)**						
Thickness of silver grating (nm)		40	50	60	70	80
Period of surface plasmon interference pattern by analytical calculation (nm)		52.8	52.8	52.8	52.8	52.8
Period of surface plasmon interference pattern by numerical simulation (nm)		52.0	52.0	52.0	52.0	52.5
Propagation length of the graphene plasmons by analytical calculation (nm)		318.7	318.7	318.7	318.7	318.7
**(c)**						
Chemical potential of upper graphene (eV)			0.3	0.4	0.5	0.6
Period of surface plasmon interference pattern by analytical calculation (nm)			39.5	45.0	48.9	51.8
Period of surface plasmon interference pattern by numerical simulation (nm)			37.0	43.3	49.3	51.0
Propagation length of the graphene plasmons by analytical calculation (nm)			237.6	271.7	295.5	313.0
**(d)**						
Chemical potential of lower graphene (eV)			0.3	0.4	0.5	0.6
Period of surface plasmon interference pattern by analytical calculation (nm)			39.5	45.0	48.9	51.8
Period of surface plasmon interference pattern by numerical simulation (nm)			37.5	43.8	47.5	50.8
Propagation length of the graphene plasmons by analytical calculation (nm)			237.6	271.7	295.5	312.9

(a) The period of graphene plasmonic interference pattern varies with the thickness of water layer in theory and simulation. (b) The relationship of the period of graphene plasmonic interference pattern with the silver grating thick in theory and simulation. (c) Period of plasmonic interference pattern changes with the chemical potential of the upper graphene. (d) Period of plasmonic interference pattern changes with the chemical potential of the lower graphene.
